# The regulation and function of acetylated high-mobility group box 1 during implantation and decidualization

**DOI:** 10.3389/fimmu.2023.1024706

**Published:** 2023-01-25

**Authors:** Yue Li, Si-Ting Chen, Yu-Ying He, Bo Li, Chen Yang, Zhen-Shan Yang, Zeng-Ming Yang

**Affiliations:** ^1^ Key Laboratory of Animal Genetics, Breeding and Reproduction in the Plateau Mountainous Region, Ministry of Education, College of Animal Science, Guizhou University, Guiyang, China; ^2^ College of Veterinary Medicine, South China Agricultural University, Guangzhou, China

**Keywords:** HMGB1, mouse, sterile inflammation, uterus, endometrium, decidualization

## Abstract

**Introduction:**

High-mobility group box 1 (HMGB1) is a non-histone nuclear protein and can be extracellularly secreted to induce sterile inflammation. Although uterine deletion of HMGB1 causes implantation and decidualization defects, how secreted HMGB1 is involved in mouse early pregnancy is still unknown.

**Methods:**

Mouse models, mouse primary endometrial cells and human endometrial cell lines were used in this study. Both immunofluorescence and Western blot were performed to show the localization and relative level of HMGB1 and acetylated HMGB1, respectively. Relative mRNA levels were analyzed by real time RT-PCR.

**Results:**

The secreted HMGB1 was detected in uterine lumen fluid in mouse periimplantation uterus. There is an obvious difference for secreted HMGB1 levels in uterine fluid between day 4 of pregnancy and day 4 of pseudopregnancy, suggesting the involvement of blastocysts during HMGB1 secretion. Trypsin is clearly detected in mouse blastocyst cavity and in the supernatant of cultured blastocysts. Trypsin significantly stimulates HB-EGF production through activating PAR2 and ADAM17. Uterine injection of PAR2 inhibitor into day 4 pregnant mice significantly reduces the number of implantation sites. HB-EGF released from luminal epithelium can induce mouse in vitro decidualization. The conditioned medium collected from trypsin-treated luminal epithelium is able to induce in vitro decidualization, which is suppressed by EGFR inhibitor. Intrauterine injection of glycyrrhizin (HMGB1 inhibitor) can significantly inhibit mouse embryo implantation. We also showed that exogenous HMGB1 released from human epithelial cells are able to induce human in vitro decidualization.

**Conclusion:**

Trypsin can induce decidualization of stromal cells via PAR2-HMGB1-ADAM17-HB-EGF from luminal epithelium.

## Introduction

High mobility group box (HMGB) family consists of *HMGB1*, *HMGB2* and *HMGB3* (1, 2). HMGB1 is a highly conserved non-histone nuclear protein (25 kDa) and shares 99% homology between rodents and humans (3, 4). The function of HMGB1 is dependent upon its location. In the nucleus, HMGB1 acts as a DNA chaperone for maintaining the structure and function of chromosomes (5). HMGB1 can also be secreted and released into extracellular environment through acetylation and other posttranslational modifications (6). HMGB1 release can be caused by stress and damaged cells (7). The secreted extracellular HMGB1 can act as a damage-associated molecular pattern (DAMP) molecule to induce sterile inflammation and immune responses through different mechanisms (5, 8).

Inflammation is essential for successful embryo implantation, pregnancy maintenance and delivery (9). Accumulating evidence indicates that the immune system at the implantation site is active and carefully controlled (10). In women successfully conceived blastocyst transfer, there is a transient and modest increase in serum pro-inflammatory cytokines 3 days after blastocyst transfer (11). ATP, uric acid, HMGB1 and cell-free fetal DNA are recently identified members of DAMP family (12). Both ATP and uric acid have been shown to play a role during decidualization (13, 14). Both *Hmgb1* mRNA and proteins are highly detected in mouse preimplantation uterus (15, 16). Conditional deletion of uterine *Hmgb1* by a *Pgr*-Cre driver shows implantation and decidualization defects (16). How secreted HMGB1 as a DAMP molecule is involved in embryo implantation and decidualization is still unknown.

Heparin-binding EGF-like growth factor (HB-EGF) is a member of the EGF family, and can bind EGF receptor (EGFR) and ErbB4 to initiate signaling (17). Similar to other EGF family members, HB-EGF is first synthesized as a membrane-anchored form (pro-HB-EGF). Pro-HB-EGF is then cleaved by metalloproteases through ectodomain shedding to form soluble HB-EGF (18). ADAM17 acts as the major convertase for epiregulin, transforming growth factor alpha, amphiregulin, and HB-EGF (19). Because Adam17-deficient mice show similar phenotype to HB-EGF-deficient mice (20), ADAM17 seems to be the major ectodomain sheddase of HB-EGF. HB-EGF is strongly expressed in the luminal epithelium at the site of blastocyst apposition on day 4 of pregnancy (21). Both conventional knockout and uterus-specific deletion of HB-EGF in mice show delayed embryo implantation and reduced litter size (22). In human aortic endothelial cells, HMGB1 can induce ADAM17 activation (23). Additionally, ADAM17 can be activated by ATP in fibroblast and by LPS in macrophages (24, 25). Whether secreted HMGB1 has an interaction with ADAM17 and HB-EGF remains undefined.

In our study, we examined acetylated HMGB1 secretion and function in mouse uterus during early pregnancy. Blastocyst-derived trypsin can induce decidualization of stromal cells via PAR2-HMGB1-ADAM17-HB-EGF from luminal epithelium.

## Materials and methods

### Animals

All animal experiments were approved by the Institutional Animal Care and Use Committee of South China Agricultural University. Mature CD1 mice were housed in a controlled environment with a 14 h light and 10 h darkness cycle. Timed mating of mice is conducted by placing females with fertile males or vasectomized males to induce pregnancy or pseudopregnancy (day 1 is the day of vaginal plug). From days 1 to 4, pregnancy was confirmed by recovering embryos from the oviducts or uteri. The implantation sites on day 5 of pregnancy was identified by tail intravenous injection of 0.1 ml of 1% Chicago sky blue (Sigma-Aldrich, St. Louis, MO).

Delayed implantation was induced by ovariectomizing pregnant mice at 08:30-09:00 on day 4 of pregnancy. Progesterone was subcutaneously injected (1 mg/mouse, Sigma-Aldrich) from days 5 to 7. Estradiol-17β (25 ng/mouse, Sigma-Aldrich) was given to progesterone-primed delayed mice to activate blastocyst implantation on day 7 of pregnancy. On day 8 of pregnancy, the mice were sacrificed to collect uteri. Implantation sites were also identified through tail intravenous injection of 0.1 ml of 1% Chicago blue dye. Delayed implantation was confirmed by flushing blastocysts from one horn of the uterus on day 8 of pregnancy and the remaining uteri were collected for further analysis.

### Intrauterine injection of inhibitors

Intrauterine injection of HMGB1 inhibitor was performed as previously described (13). At 09:00 on the day 4 of pregnancy, two uterine horns of each mouse were slowly injected with Glycyrrhizin (3 μl per uterine horn, 100 μM in saline, NSC 167409, Selleck), a specific HMGB1 inhibitor. In control group, two uterine horns of each mouse were injected with an equal volume of normal saline (3 μl per uterine horn).

### Isolation and treatment of mouse uterine luminal epithelial cells

The mouse luminal epithelial cells were isolated as previously described (26). Briefly, the uteri were digested with sterile HBSS containing 0.3% trypsin and 6 mg/ml dispase for 1.5 h at 4 °C followed by 30 min at room temperature and 10 min at 37 °C. The luminal epithelial cells were cultured in DMEM/F-12 medium (Sigma-Aldrich) containing 10% heat-inactivated fetal bovine serum (FBS, Invitrogen, USA). The epithelial cells were treated with recombinant HMGB1 (Sigma-Aldrich).

### Isolation and in vitro decidualization culture of mouse uterine stromal cells

Stromal cells were isolated from mouse uteri as previously described (13). In brief, mouse uteri on day 4 of pregnancy were digested with 1% trypsin (0458, AMRESCO) and 6 mg/ml dispase (0494207801, Roche) in Hanks’ balanced salt solution (H4891, Sigma-Aldrich). After luminal epithelial cells were removed by washing, the remaining uteri were incubated with 0.15 mg/ml collagenase I (17100017, Gibco). The isolated stromal cells were cultured in DMEM/ F12 (D2906, Sigma-Aldrich) containing 10% charcoal-treated FBS (Biological Industries, Israel). In vitro decidualization of endometrial stromal cells was induced with 1 mM progesterone and 10 nM estradiol-17β. Mouse stromal cells were treated with 0, 2, 10, and 50 ng/ml HMGB1 for 24 h, respectively.

### Culture of human stromal cells and Ishikawa epithelial cells

Human uterine stromal cells and Ishikawa epithelial cells were obtained from ATCC and cultured in DMEM/F12 supplemented with 10% FBS. *In vitro* decidualization of human stromal cells was induced by treatment of the cells with 1 μM medroxyprogesterone (MPA, Sigma-Aldrich, St. Louis, MO), and 500 μM cyclic adenosine monophosphate (cAMP, Sigma-Aldrich) in DMEM/F12 supplemented with 2% cFBS as previously described (27).

### Co-culture of uterine epithelial cells and stromal cells

After uterine epithelial cells were cultured and treated, the conditioned medium was collected from cultured epithelial cells. The uterine stromal cells were cultured in the conditioned medium from epithelial cells and induced for in vitro decidualization.

### Immunofluorescence

Immunofluorescence was performed as previously described (26). Briefly, paraffin sections were hydrated, permeabilized with 1% Triton X-100 in PBS for 10 min, and blocked with 10% horse serum at 37°C for 1 h. Sections were then incubated with anti-Acetyl-HMGB1 (1:1000, A16002, Abclonal Technology) overnight at 4°C. The same concentration of matched non-specific IgG was used for negative control. After washing in PBS, sections were incubated with the corresponding secondary antibody conjugated with FITC for 30 min. Sections were counter-stained with propidium iodide (PI, Sigma-Aldrich) and mounted for fluorescence analysis. Images were obtained by confocal microscope (Leica TCS SP8) with Leica Application Suite X.

### Real-time RT-PCR

Total RNAs were isolated with TRIzol reagent kit (T9109, TaKaRa) and reverse transcribed with PrimeScript reverse transcriptase reagent kit (R233, Vazyme, Nanjing, China) according to the manufacturer’s instructions. RT-qPCR was performed using SYBR (Q311, Vazyme, Nanjing, China) on a Bio-Rad CFX96 Touch™ Real-Time System thermocycler using gene-specific primers. Gene expression was analyzed after Ct values were normalized to the Rpl7 housekeeping gene. Primers used in this study was listed in **Table 1**.

**Table 1:** Primer sequences used in this study.

Gene	Species	Sequence (5’-3’)	Application	Accession Number	Product size
*IGFBP1*	Human	CCAAACTGCAACAAGAATG GTAGACGCACCAGCAGAG	RT-qPCR	NM_001013029	87 bp
*Lif*	Mouse	AAAAGCTATGTGCGCCTAACA GTATGCGACCATCCGATACAG	RT-qPCR	NM_008501	98 bp
*Prl8a2*	Mouse	AGCCAGAAATCACTGCCACT TGATCCATGCACCCATAAAA	RT-qPCR	NM_010088	119 bp
*PRL*	Human	AAGCTGTAGAGATTGAGGAGCAAA TCAGGATGAACCTGGCTGACTA	RT-qPCR	NM_000948	76 bp
*Rpl7*	Mouse	GCAGATGTACCGCACTGAGATTC ACCTTTGGGCTTACTCCATTGATA	RT-qPCR	NM_011291.5	129 bp
*RPL7*	Human	CTGCTGTGCCAGAAACCCTT TCTTGCCATCCTCGCCAT	RT-qPCR	NM_000971	194 bp

### Western blot

Western blot was performed as previously described (26). Cultured cells were collected for protein extraction. Protein lysates were separated by SDS-PAGE. After proteins were transferred onto PVDF membranes, membranes were incubated with each primary antibody, α-Tubulin (1:1000, 2144, Cell Signaling Technology), p-Stat3 (1:1000, 9145, Cell Signaling Technology), STAT3 (1:1000, sc-7179, Santa Cruz Biotechnology), HB-EGF (1:500, sc-28908, Santa Cruz Biotechnology), Acetyl-HMGB1 (1:1000, A16002, Abclonal Technology), or ADAM17 (1:1500, A0821, Abclonal Technology) overnight at 4°C. After membranes were incubated with the corresponding HRP-conjugated secondary antibody for 1 h, signals were detected through ECL Chemiluminescent kit (Millipore).

### Vacuum freeze drying

Mouse uteri were flushed with 0.5 ml of normal saline. The uterine fluid was dialyzed in distilled water at room temperature for 2 h and dried in a vacuum freeze drier at -80°C for 24 h.

### Measurement of mouse trypsin protein level

Mouse blastocysts were collected from day 4 pregnant mice at 9:00 by flushing uterine lumen with M2 medium (Sigma-Aldrich). Total 100 blastocysts were added into 200 μl of M2 medium, frozen and thawed in liquid nitrogen twice, and centrifugated at 13,000 rpm for 10 min. After centrifugation, the supernatant was collected for measuring trypsin. This treatment was independently repeated 3 times.

Additionally, 100 mouse blastocysts collected from day 4 pregnant mice were cultured in 200 μl of M2 medium in CO2 incubator at 37 °C for 6 h. The supernatant from cultured blastocysts was collected for measuring trypsin level. This treatment was independently repeated 3 times.

Trypsin ELISA kit (Camilo biological, 2M-KMLJM220814m, Nanjing, China) was used to measure trypsin level. This kit's sensitivity is greater than 0.5 ng/ml. Total 200 μl sample was used to measure trypsin as per the manufacture's instruction. The absorbance was measured at 450 nm with Biotek microplate reader (ELX808).

### Statistics

Data were presented as means ± SEMs. All the treatments were repeated at least 3 times. Statistical analysis was performed with an unpaired Student’s t-test. p<0.05 was considered statistically significant.

## Results

### Localization and secretion of acetylated HMGB1 in mouse uterus during early pregnancy

**Figure 1:**
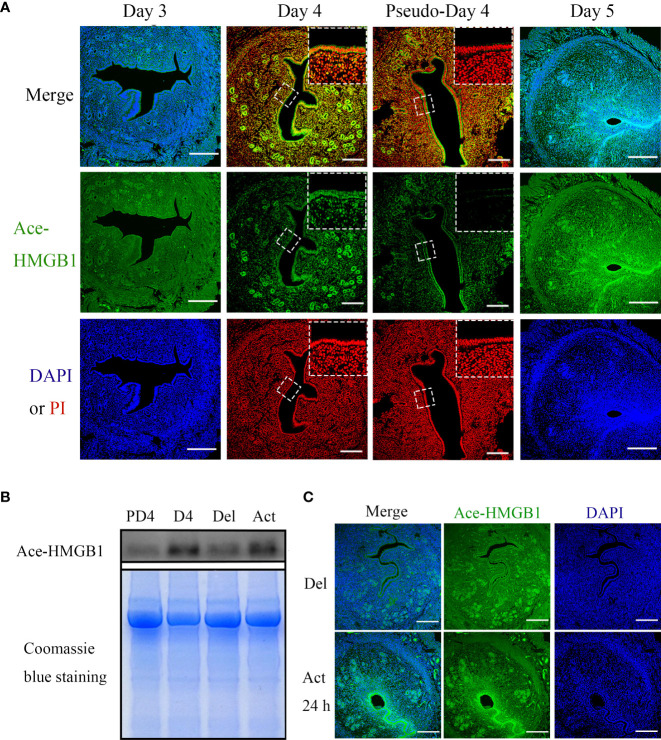
The immunofluorescence and protein levels of Ace-HMGB1 in mouse uterus during early pregnancy. **(A)** Acetylated HMGB1 immunofluorescence in mouse uteri on days 3, 4 and 5 of pregnancy, and on day 4 of pseudopregnancy. Scale bar=250 μm. **(B)** The protein levels of acetylated HMGB1 in uterine fluid collected on day 4 of pregnancy and day 4 of pseudopregnancy, and in uterine lumen fluid collected from mouse uterus under delayed implantation and 12 h after estrogen activation. The Coomassie blue staining of uterine fluid was served as a loading control. **(C)** The acetylated HMGB1 immunofluorescence in mouse uterus under delayed implantation and estrogen activation. Scale bar=250 μm.

Because HMGB1 can only be secreted following acetylation and acetylated HMGB1 can act as a damage-associated molecular pattern (DAMP) molecule to induce sterile inflammation and immune responses through different mechanisms (5, 8), we mainly focused on acetylated HMGB1 during early pregnancy. From days 1 to 3, there was no detectable acetylated HMGB1 immunofluorescence in mouse uteri (**Figure 1A**). On day 4, acetylated HMGB1 immunofluorescence was mainly localized in luminal and glandular epithelium. Compared to day 4 of pregnancy, acetylated HMGB1 immunofluorescence was weakly seen in uterine epithelium and stroma on day 4 of pseudopregnancy (**Figure 1A**). Western blot also showed that the protein level of acetylated HMGB1 in the uterine fluid on day 4 of pregnancy was significantly higher than that on day 4 of pseudopregnancy (**Figure 1B**). On day 5, acetylated HMGB1 immunofluorescence was mainly localized in luminal epithelium and the stroma around the implanting blastocyst (**Figure 1A**).

Because acetylated HMGB1 immunofluorescence was different between day 4 of pregnancy and day 4 of pseudopregnancy, acetylated HMGB1 immunofluorescence was further examined under delayed implantation. Under delayed implantation, acetylated HMGB1 signal was weakly seen in glandular epithelium. After delayed implantation was activated by estrogen for 24 h, acetylated HMGB1 immunofluorescence was strongly observed in luminal epithelium and the luminal epithelium surrounding implanting blastocyst (**Figure 1C**).

### Embryonic regulation of acetylated-HMGB1 secretion from mouse uterine luminal epithelial cells

**Figure 2:**
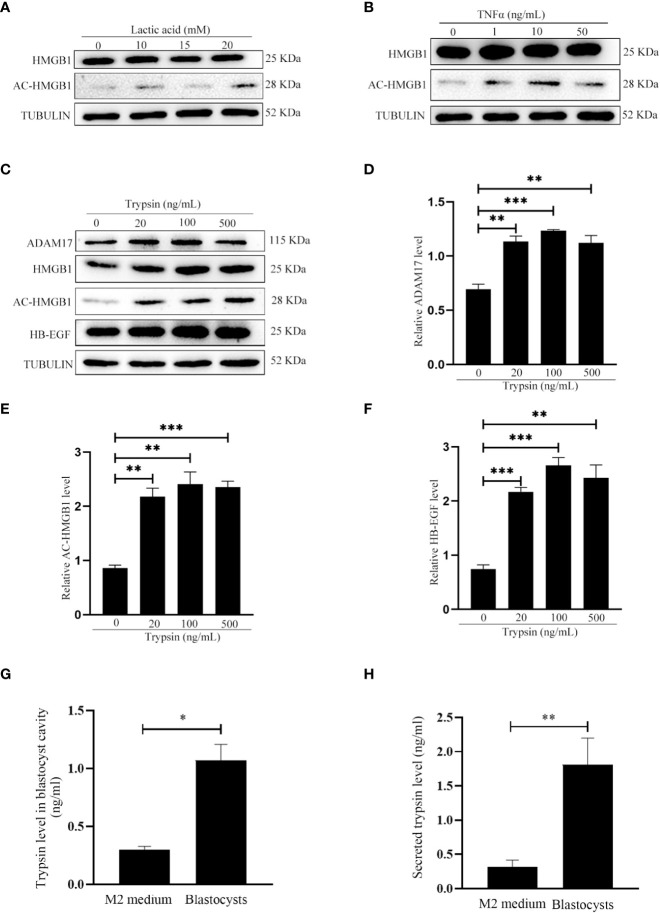
Effects of lactate, TNFα and trypsin on the levels of HMGB1, acetylated HMGB1, ADAM17 and HB-EGF in cultured mouse epithelial cells. **(A)** Western blot analysis of the protein levels of HMGB1 and acetylated HMGB1 after epithelial cells were treated with lactic acid for 15 min. **(B)** Western blot analysis of the protein levels of HMGB1 and acetylated HMGB1 after epithelial cells were treated with TNFα for 15min. **(C)** Western blot analysis of the protein levels of ADAM17, HMGB1, acetylated HMGB1 and HB-EGF after epithelial cells were treated with trypsin for 15 min. **(D-F)** Relative quantitative analysis on the protein levels of ADAM17, acetylated HMGB1 and HB-EGF in **Figure 2E**, respectively. **(G)** The trypsin level in blastocyst cavity from 100 mouse blastocysts. **(H)** The trypsin level in the supernatant after 100 mouse blastocysts were cultured in 200 μl of M2 medium for 6 h. **p < 0.01, ***p < 0.001.

Because acetylated HMGB1 level was different between pregnancy and pseudopregnancy on day 4, and between delayed implantation and activation, we assumed that blastocyst should play a role on acetylated HMGB1 secretion. Previous studies indicated that mouse blastocysts secrete lactic acid, trypsin and TNFα (26, 28, 29). When mouse uterine luminal epithelial cells were treated with lactic acid or TNFα, there were slight effects on the levels of HB-EGF and acetylated HMGB1 (**Figures 2A, B**). However, the levels of acetylated HMGB1, HB-EGF and ADAM17 were significantly increased after epithelial cells were treated with trypsin (**Figures 2C–F**). In mouse blastocysts on day 4 of pregnancy, a high level of trypsin protein was detected in blastocyst cavity (**Figure 2G**). Furthermore, a high level of secreted trypsin protein was measured in the cultured medium after mouse blastocysts on day 4 of pregnancy were cultured in vitro for 6 h (**Figure 2H**).

### Effects of HMGB1 on mouse implantation and in vitro decidualization

**Figure 3:**
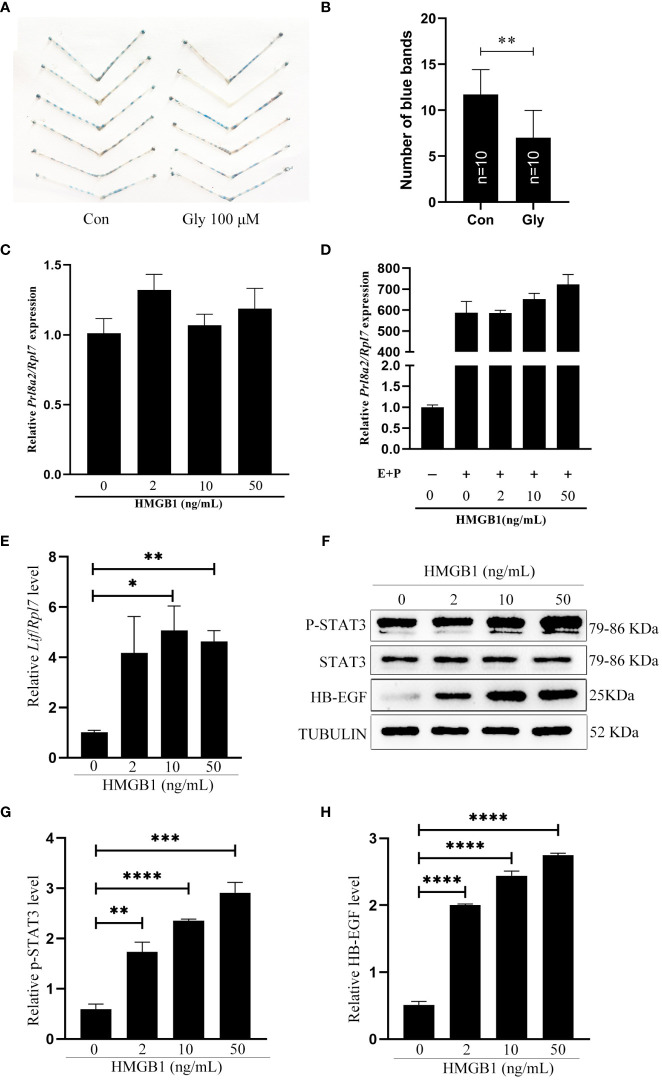
Effects of recombinant HMGB1 proteins on mouse implantation and decidualization. **(A)** The morphology of mouse uteri on day 5 of pregnancy after day 4 pregnant mice were intraluminally injected with 3 μl of saline or 100 μM Glycyrrhizin in saline at 09:00. **(B)** The number of implantation sites (blue bands) on day 5 of pregnancy after day 4 pregnant mice were intraluminally injected with 3 μl of saline or 100 μM Glycyrrhizin in saline at 09:00. The implantation sites were showed by injecting 0.1 ml of 1% Chicago sky blue through tail vein. **(C)** The *Prl8a2* mRNA level after mouse stromal cells were treated with 0, 2, 10 and 50 ng/ml HMGB1 for 24 h, respectively. **(D)** The *Prl8a2* level after mouse stromal cells were treated with 0, 2, 10 and 50 ng/ml HMGB1 for 24 h under in vitro decidualization, respectively. **(E)** The *Lif* mRNA level after mouse epithelial cells were treated with 0, 2, 10 and 50 ng/ml HMGB1 for 15 min. **(F)** Western blot analysis on the protein levels of p-Stat3, STAT3, and HB-EGF after mouse epithelial cells were treated with HMGB1 for 15 min. **(G, H)** The relative quantification of p-STAT3 and HB-EGF protein levels in **Figure 3F**. *p < 0.05, **p < 0.01, ***p < 0.001, ****p < 0.0001.

Because acetylated HMGB1 was strongly detected in uterine fluid and epithelial cells on day 4 of pregnancy, we were wondering whether HMGB1 is involved in mouse embryo implantation. When 3μl Glycyrrhizin (100 μM), a specific HMGB1 inhibitor, was injected into uterine lumen at 09:00 on day 4 of pregnancy, the number of implantation sites on day 5 of pregnancy was significantly reduced compared to control (**Figures 3A, B**), confirming the importance of HMGB1 during early pregnancy. When mouse uterine stromal cells were treated with HMGB1 directly or under in vitro decidualization, the mRNA level of *Prl8a2*, a reliable marker for mouse in vitro decidualization (30), was slightly increased, but not significant (**Figures 3C, D**). Therefore, we speculated whether acetylated HMGB1 might affect decidualization indirectly through luminal epithelium.

### Effects of exogenous HMGB1 on mouse uterine receptivity

LIF, p-STAT3 and HB-EGF are recognized markers for mouse uterine receptivity (21, 31, 32). When mouse epithelial cells were treated with HMGB1 for 15 min, *Lif* mRNA level was significantly induced compared to control (**Figure 3E**). Western blot results showed that both p-STAT3 and HB-EGF protein levels were also significantly and dose-dependently increased after epithelial cells were treated with HMGB1 (**Figures 3F–H**).

### Treatment of mouse epithelial cells with FSLLRY-NH_2_ and TAPI-1

**Figure 4:**
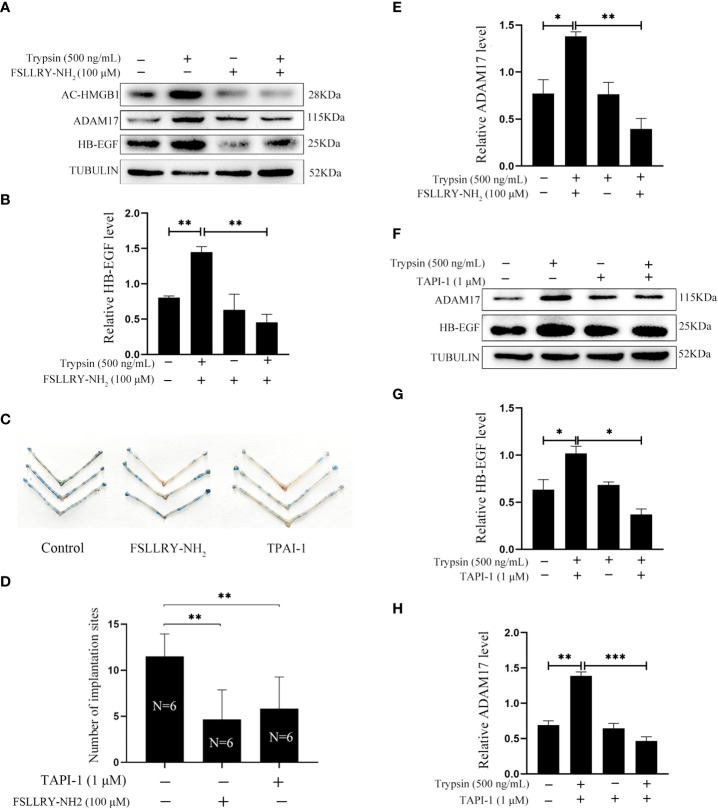
Effects of PAR2 inhibitor and ADAM17 inhibitor on the protein levels of acetylated HMGB1, ADAM17 and HB-EGF in cultured mouse epithelial cells. **(A)** Western blot analysis on the protein levels of acetylated HMGB1, ADAM17 and HB-EGF after mouse epithelial cells were pretreated with FSLLRY-NH_2_ (100 μM, PAR2 inhibitor) for 24 h and further treated with trypsin for 15 min in the absence or presence of FSLLRY-NH_2_. **(B)** The relative HB-EGF level in **Figure 4A**. **(C)** The morphology of mouse uteri on day 5 of pregnancy after day 4 pregnant mice were intraluminally injected with 3 μl of saline, 100 μM of FSLLRY-NH_2_ in saline or 1 μM TAPI-1 in saline at 09:00. **(D)** The number of implantation sites (blue bands) on day 5 of pregnancy after 3 μl of saline, 100 μM FSLLRY-NH_2_ in saline, or 1 μM TAPI-1 in saline were intraluminally injected into pregnant mice at 09:00. By injecting 0.1 ml of 1% Chicago sky blue through the tail vein, the implantation sites were counted. **(E)** The relative ADAM17 in **Figure 4A**. **(F)** Western blot analysis on the protein levels of ADAM17 and HB-EGF after mouse epithelial cells were pretreated with TAPI-1 (1 μM, ADAM17 inhibitor) for 30 min and further treated with trypsin for 15 min in the absence or presence of TAPI-1. **(G, H)** Relative quantitation on the protein levels of HB-EGF and ADAM17 in **Figure 4F**. *p < 0.05, **p < 0.01, ***p < 0.001.

Trypsin is a member of serine proteinase family and mainly activate proteinase-activated receptor 2 (PAR2) (33–35). When mouse epithelial cells were treated with 500 ng/ml trypsin for 15 min, the protein levels of acetylated HMGB1 and HB-EGF were significantly increased, which were significantly abrogated by FSLLRY-NH_2_, a specific PAR2 inhibitor (**Figures 4A, B**). On day 5 of pregnancy, the number of implantation sites was significantly reduced after 3μl of 100 μM FSLLRY-NH_2_ were injected into uterine lumen on day 4 of pregnancy (**Figures 4C, D**), suggesting that blastocysts-derived trypsin might be inhibited by FSLLRY-NH_2_. ADAM17 is the major ectodomain sheddase of HB-EGF Figure (20, 36). The trypsin-stimulated increase of ADAM17 was suppressed by FSLLRY-NH_2_ (**Figures 4A, E**). Additionally, trypsin-induced increase of ADAM17 and HB-EGF protein levels were significantly abrogated by TAPI-1, a specific inhibitor of ADAM17 (**Figures 4F–H**). The number of implantation sites on day 5 of pregnancy was significantly decreased after 3 μl of 1 μM TAPI-1 were injected into the uterine lumen on day 4 of pregnancy (**Figures 4C, D**). These results indicated that trypsin-induced HB-EGF production from mouse luminal epithelium through PAR2-HMGB1-ADAM17 pathway.

### Effects of epithelium-derived HB-EGF on in vitro decidualization of mouse stromal cells

**Figure 5:**
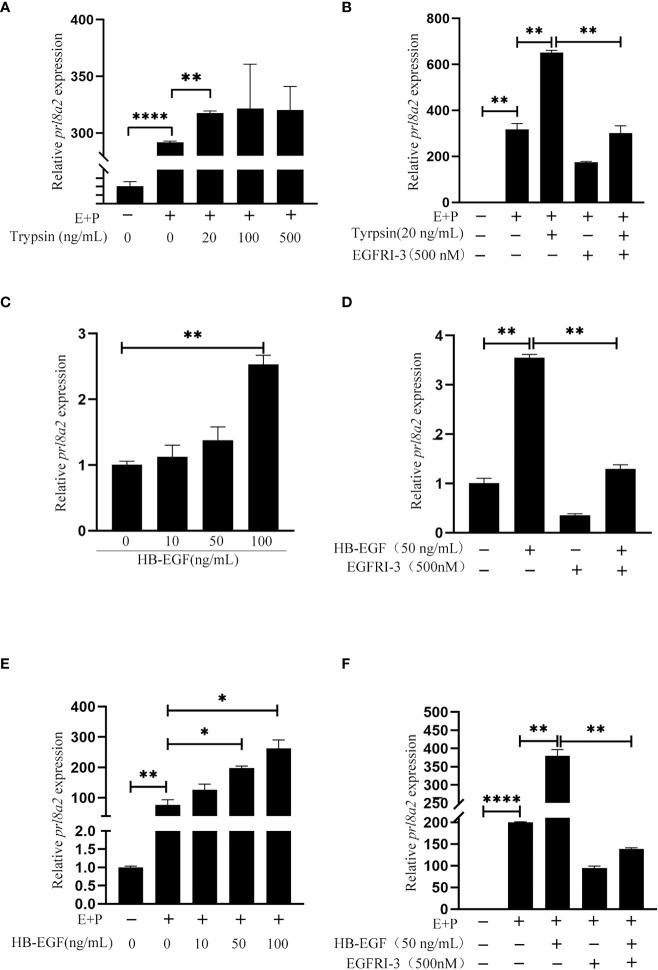
Effects of the conditioned medium from trypsin-treated epithelial cells and HB-EGF on mouse in vitro decidualization. **(A)** The *Prl8a2* mRNA level after stromal cells were treated with the conditioned medium from trypsin-treated epithelial cells under in vitro decidualization. **(B)** Effects of EGFRI-3 (500 nM, EGFR inhibitor) on the *Prl8a2* mRNA level after stromal cells were treated with the conditioned medium from trypsin-treated epithelial cells. **(C)** The *Prl8a2* mRNA level after mouse stromal cells were treated with different concentrations of HB-EGF. **(D)** Effects of EGFRI-3 on *Prl8a2* mRNA levels after stromal cells were treated with HB-EGF. **(E)** The *Prl8a2* mRNA level after stromal cells were treated with HB-EGF under in vitro decidualization. **(F)** Effects of EGFRI-3 on *Prl8a2* mRNA level after stromal cells were treated with HB-EGF under in vitro decidualization. The mRNA level of *Prl8a2* was normalized to ribosomal protein 7 (*Rpl7*) mRNA level. *p < 0.05, **p < 0.01, ****p < 0.0001.

In order to examine whether epithelium-derived HB-EGF has an effect on in vitro decidualization of mouse stromal cell, mouse epithelial cells were treated with trypsin for 15 min. The conditioned medium collected from trypsin-treated epithelial cells were used to treat mouse stromal cells. Compared to control, *Prl8a2* mRNA level was significantly increased by the medium conditioned by trypsin-treated mouse epithelial cells (**Figure 5A**). When stromal cells were pretreated with EGFRI-3, a specific EGFR inhibitor, EGFRI-3 significantly suppressed the increase of *Prl8a2* mRNA level stimulated by trypsin-treated conditioned medium (**Figure 5B**).

When mouse stromal cells were directly treated with HB-EGF or treated with HB-EGF under in vitro decidualization, *Prl8a2* mRNA level was significantly increased (**Figures 5C, E**), which was significantly abrogated by EGFRI-3 (**Figures 5D, F**), suggesting that HB-EGF stimulates mouse decidualization through EGFR.

### Effects of extracellular HMGB1 on human uterine stromal cells and epithelial cells

**Figure 6:**
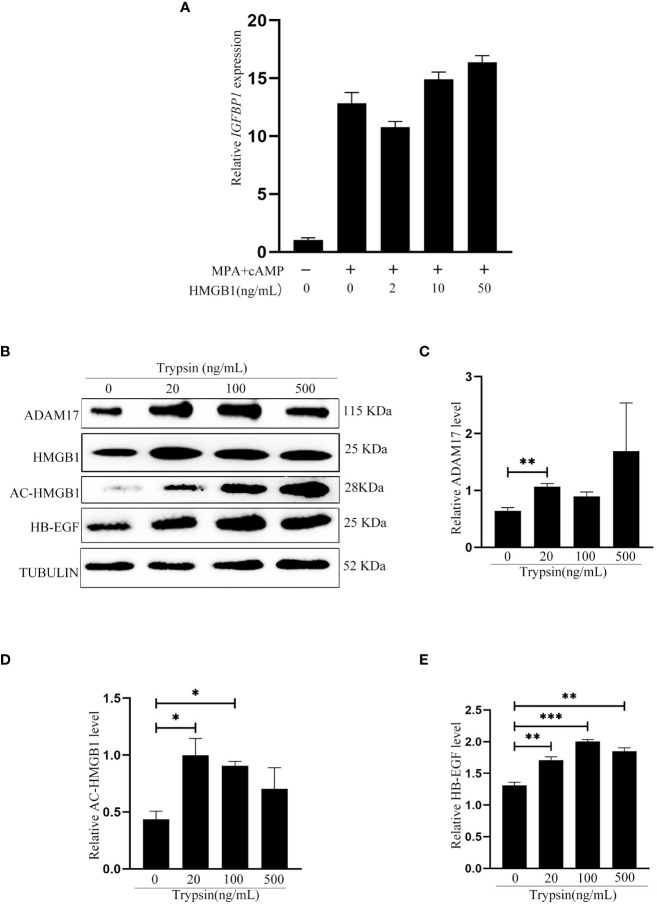
Effects of exogenous HMGB1on human decidualization and human uterine epithelial cells. **(A)** Effects of exogenous HMGB1 on IGFBP1 mRNA level under in vitro decidualization. **(B)** Western blot analysis on the protein levels of ADAM17, HMGB1, Ace-HMGB1 and HB-EGF after human Ishikawa epithelial cells were treated with trypsin. **(C-E)** The relative protein levels of ADAM17, acetylated HMGB1 and HB-EGF in **Figure 6B**, respectively. *p < 0.05; **p < 0.01, ***p < 0.001.

After we showed that exogenous HMGB1 stimulated mouse decidualization via luminal epithelium, we were wondering whether HMGB1 has a similar effect on human decidualization. Similarly, exogenous HMGB1 had no detectable effect on in vitro decidualization of human stromal cells (**Figure 6A**). When human Ishikawa epithelial cells were treated with trypsin, the protein levels of acetylated HMGB1, ADAM17 and HB-EGF were significantly increased (**Figures 6B–E**).

### Trypsin regulation on human HB-EGF via PAR2 and ADAM17

**Figure 7:**
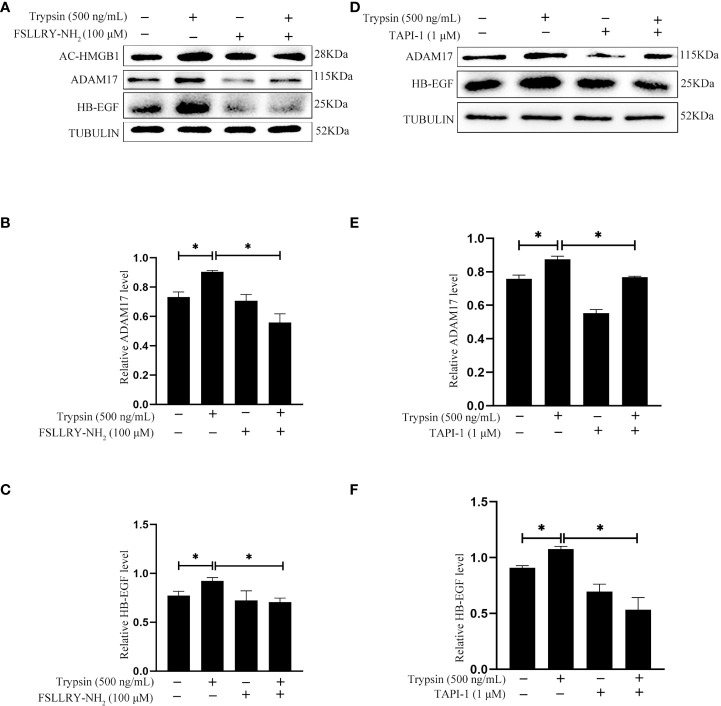
Effects of PAR2 inhibitor and ADAM17 inhibitor on the protein levels of acetylated HMGB1, ADAM17 and HB-EGF after human Ishikawa cells were treated with 500 ng/ml trypsin. **(A)** Western blot analysis on the protein levels of acetylated HMGB1, ADAM17 and HB-EGF when Ishikawa cells were pretreated with 100 μM FSLLRY-NH_2_ for 24 h and further treated with 500 ng/ml trypsin in the absence or presence of FSLLRY-NH_2_. **(B, C)** The relative protein levels of ADAM17 and HB-EGF in **Figure 7A**. **(D)** Western blot analysis on the protein levels of ADAM17 and HB-EGF when Ishikawa cells were pretreated with 1 μM TAPI-1 for 30 min and further treated with 500 ng/ml trypsin for 15 min. **(E, F)** The relative protein levels of ADAM17 and HB-EGF in **Figure 7D**.*, p <0.05.

Trypsin is a member of serine proteinase family and mainly activate proteinase-activated receptor 2 (PAR2) (33–35). When human Ishikawa epithelial cells were treated with 500 ng/ml trypsin for 15 min, the protein levels of acetylated HMGB1 and HB-EGF were significantly increased, which were significantly abrogated by FSLLRY-NH_2_, a specific PAR2 inhibitor (**Figures 7A–C**). ADAM17 is the major ectodomain sheddase of HB-EGF (20, 36). Additionally, trypsin-induced increase of ADAM17 and HB-EGF protein levels were significantly abrogated by TAPI-1, a specific inhibitor of ADAM17 (**Figures 7D–F**). These results indicated that trypsin induced HB-EGF production from human endometrial epithelium through PAR2-HMGB1-ADAM17 pathway.

### Effects of epithelium-derived HB-EGF on in vitro decidualization of human stromal cells

**Figure 8:**
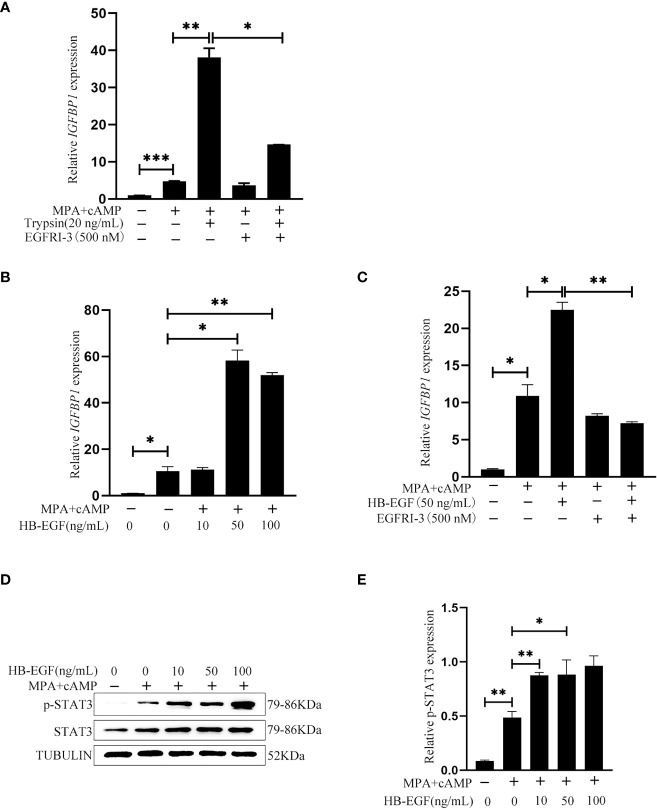
Effects of the conditioned medium from trypsin-treated Ishikawa cells and HB-EGF on human in vitro decidualization. **(A)** Effects of the conditioned medium from trypsin-treated Ishikawa cells on IGFBP1 mRNA level after stromal cells were cultured with the conditioned medium plus EGFRI-3 under in vitro decidualization for 24 h **(B)** The *IGFBP1* mRNA level after human endometrial cells were treated with HB-EGF under in vitro decidualization. **(C)** Effects of EGFR inhibitors on IGFBP1 mRNA level when human stromal cells were treated with HB-EGF under in vitro decidualization. **(D)** Western blot analysis on p-STAT3 and STAT3 protein levels when human stromal cells were treated with HB-EGF under in vitro decidualization. **(E)** The relative protein level of p-STAT3 in **Figure 8D**. *, p<0.05; **, p<0.01.

After human Ishikawa epithelial cells were treated with trypsin, the medium was collected to culture stromal cells for inducing in vitro decidualization. IGFBP1, a reliable marker of human in vitro decidualization (37), was significantly induced by the medium conditioned by trypsin-treated epithelial cells, which was significantly abrogated by EGFRI-3, a specific EGFR inhibitor (**Figure 8A**). When human stromal cells were treated with 10, 50 and 100 ng/ml HB-EGF under in vitro decidualization, *IGFBP1* mRNA level was significantly increased by 50 and 100 ng/ml HB-EGF (**Figure 8B**), which was significantly suppressed by EGFRI-3 (**Figure 8C**). Additionally, the protein level of p-STAT3 under in vitro decidualization was significantly stimulated by HB-EGF (**Figures 8D, E**).

## Discussion

In this study, the level of acetylated HMGB1 in uterine luminal fluid in day 4 pregnant mouse uterus is significantly higher than that in day 4 pseudopregnancy uterus. Compared to delayed implantation, the level of acetylated HMGB1 in luminal fluid is also significantly higher after delayed implantation is activated by estrogen. Meanwhile, the immunofluorescence of acetylated HMGB1 in luminal epithelium also shows a similar pattern to the acetylated HMGB1 level in luminal fluid. These results strongly suggest the involvement of blastocyst in the secretion of acetylated HMGB1. Trypsin, lactic acid and TNFα are synthesized and secreted by mouse blastocysts (26, 28, 29). When mouse uterine epithelial cells are treated with trypsin, the secreted level of acetylated HMGB1 is higher than treatment with lactic acid or TNFα, suggesting that trypsin should be a dominant blastocyst-derived stimulus. In our study, trypsin is detected both in mouse blastocyst cavity and in the supernatant of cultured blastocysts. However, trypsin treatment significantly stimulates the secretion of acetylated HMGB1 from human Ishikawa cells. Four Proteinase-activated receptors (PAR1-4) have been identified and differ in the specific substrates and localization (38). During the menstrual cycle, PAR2 mRNA is expressed in human endometrial tissues and can be activated by trypsin (33–35). When epithelial cells are treated with FSLLRY-NH_2_, a PAR2 inhibitor, trypsin-stimulated HMGB1 secretion is significantly abrogated. Our result also indicated that the number of implantation sites is significantly decreased after FSLLRY-NH_2_ is intraluminally injected into day 4 pregnant mice. The embryo implantation in mice and rats are inhibited by AEBSF, a specific inhibitor for serine proteinase family (39, 40). These data indicated that HMGB1 secretion from uterine luminal epithelium should be controlled by trypsin-PAR2 pathway.

Our study indicated that HMGB1 promotes decidualization through ectodomain sheddase of HB-EGF released from luminal epithelium although treatment of stromal cells with HMGB1 has little effects on in vitro decidualization. Mouse embryo implantation is obviously suppressed by uterine injection of Glycyrrhizin, a specific HMGB1 inhibitor. Furthermore, HMGB1 treatment significantly increases the level of LIF and p-STAT3 in luminal epithelium, markers for uterine receptivity (31, 32). HMGB1 is strongly expressed in mouse perimplantation uterus (15, 16). Uterine deletion of HMGB1 causes implantation and decidualization abnormality (16). Our data suggest that secreted HMGB1 as an inflammation factor plays a key role during embryo implantation and decidualization. A previous study also showed the beneficial effect of HMGB1 on mouse decidualization (15). However, the increased expression of HMGB1 in the implantation phase endometrium is related to recurrent implantation failure (41). HMGB1 expression is significantly increased in villi and decidua in unexplained recurrent spontaneous abortion (URSA) group compared with those in the control group. In the URSA group, HMGB1 was co-localized with the CD45-labeled immune cells (42). The accumulating evidence also indicates that physiological inflammation should be essential for successful embryo implantation and decidualization (9). Our previous studies also showed that ATP and uric acid, two marker molecules of sterile inflammation, can promote decidualization (13, 14). A recent study showed that human blastocysts are able to modulate the inflammation response in maternal decidua (43). In our study, blastocyst-derived trypsin is able to stimulate HMGB1 release, suggesting that the quality of blastocyst is essential for proper inflammation response in decidua. Although our study showed that the secretion of HMGB1 is embryo-dependent, mouse decidualization can also be artificially induced by mechanic injury or inflammatory stimuli (44). Different from mice, human decidualization spontaneously occurs and is initiated by progesterone and cAMP (45). When tissues or cells are injured or damaged under stress, there is a release of sterile inflammation molecules, including ATP, uric acid and HMGB1 (46). Our previous studies identified that both ATP and uric acid are released during embryo implantation and are able to induce in vitro decidualization in mice and humans (13, 14).

HMGB1 is a position-dependent multifunctional protein (47). HMGB1 secretion is mainly controlled through acetylation (48). Human in vitro decidualization is significantly stimulated by trichostatin A (TSA), a specific HDAC inhibitor (49). These data also indicate that the acetylation of HMGB1 should be essential to decidualization.

Our data indicated that HMGB1 stimulates HB-EGF production through ADAM17. Trypsin-induced increase of mature HB-EGF in epithelial cells is significantly suppressed by TAPI-1, a specific inhibitor of ADAM17. Trypsin-stimulated increase of ADAM17 and mature HB-EGF is also abrogated by FSLLRY-NH_2_, a specific inhibitor of PAR2. Trypsin activity has been identified in cultured medium of mouse and human blastocysts (29, 50). In human uterine epithelial cells, trypsin triggers intracellular calcium signaling through binding PAR2 (51). In mouse uterine epithelial cells, trypsin stimulates calcium signaling for PGE2 release by activating epithelial sodium channel (52). In human endometrium, ADAM17 immunofluorescence is mainly localized in luminal and glandular epithelium of the receptive phase human endometrium (53). We also showed that mouse and human decidualization is induced by HB-EGF. HB-EGF is strongly expressed in mouse luminal epithelium at the site of blastocyst apposition on day 4 of pregnancy (21). Previous studies also indicated that HB-EGF is beneficial for both mouse and human in vitro decidualization (54, 55). Both conventional and uterine deletion of mouse HB-EGF causes implantation and decidualization defects (22).

## Conclusion

**Figure 9:**
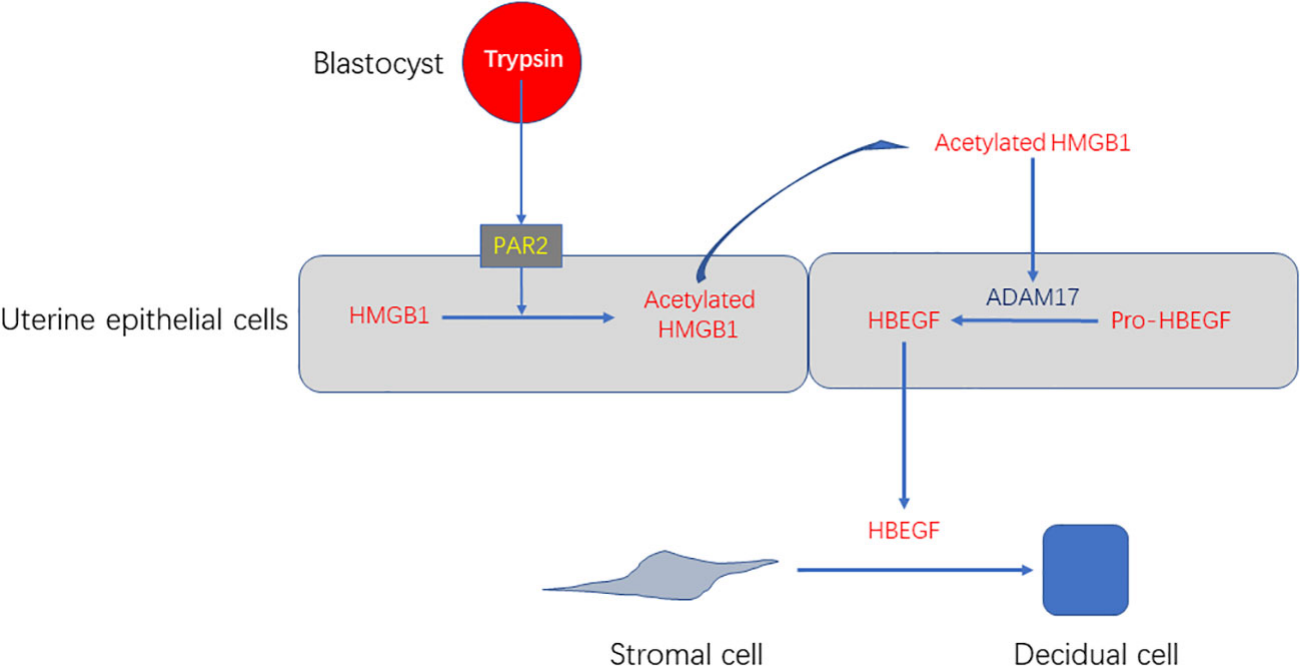
A working model showing that blastocyst-derived trypsin induces decidualization of stromal cells through PAR2-HMGB1-ADAM17-HB-EGF.

In this study, we show that trypsin can induce decidualization of stromal cells through PAR2-HMGB1-ADAM17-HB-EGF in luminal epithelium in both mice and humans (**Figure 9**). Although excessive inflammation often causes infertility, a physiological level of exogenous HMGB1 should be beneficial for mouse and human decidualization.

## Data availability statement

The original contributions presented in the study are included in the article/supplementary material. Further inquiries can be directed to the corresponding author.

## Ethics statement

The animal study was reviewed and approved by Institutional Animal Care and Use Committee of South China Agricultural University.

## Author contributions

Design experiment: YL, ZMY. Experiments performed: YL, STC, HYY, BL, ZSY, CY. Data analysis: LY, ZSY, CY, ZMY. Writing - manuscript: YL, ZMY. Writing - review & editing: YL, ZMY. All authors read and approved the final manuscript.
